# Our Children’s Teachers

**Published:** 1859-12

**Authors:** 


### HALL'S JOURNAL OF HEALTH.

OUR LEGITIMATE SCOPE IS ALMOST BOUNDLESS: FOR WHATEVER BEGETS PLEASURABLE AND HARMLESS FEELINGS, PROMOTES HEALTH; AND WHATEVER INDUCES DISAGREEABLE SENSATIONS, ENGENDERS DISEASE.

*We aim to show how Disease may be avoided, and that it is best, when sickness comes, to take no Medicine without consulting an educated Physician.*

VOL. VI.] DECEMBER, 1859. [No. 12.

### OUR CHILDREN'S TEACHERS.

A seventy-five cent volume called "Uses and Abuses of Air," has never yielded its talented author a dollar's profit, and yet it is one of the most practically useful books on that subject which we have seen. But it is not the taste of the times to patronize the books, papers, magazines, which are best calculated to promote public good, and advance the true and permanent interests of the masses. This is the reign of cheap literature. The great patronized now is, pictorial magazines, weeklies filled with wishey-washey love tales, or stories of the terrible, with "horrible" headings. Scarce anything attracts attention now, short of a "midnight murder," or a "shocking catastrophe." "Eugene Sue," "Hot Corn," and "Uncle Tom," with their exaggerated imaginings, sell by the scores of thousands, pandering to the passions in morals, or in politics, stirring up brother to fiercer fight against his brother, or stimulating prurient searches after things which ought to remain unexposed; while better books, books whose aim and end is to instruct the masses as to the preservation of health, the maintenance of good constitutions; which seek to invite the young to profitable industries, to elevating amusements, and refining associations, cumber the shelves of the philanthropic bookseller for years together. The Religious Weekly made up of articles fit to be read in any assembly, purifying the heart, instructing the conscience, expanding the intellect, and moulding the morals for a higher sphere, leads a gasping life, existing from day to day; while the flash journal, filled with the most inane trash in part, and in other part, feeding the mirth of the multitude with stale jests or newer "inventions" at the expense of the rich, the religious and the good, these count their quarter of a million weekly sales. This will be the case just as long as the newspaper supplants the Bible, just as long as parents commit the religious instruction of their children to others, almost wholly to the hired governess, the Sunday school teacher and the occasional lecture of common instructors, too seldom turning to the original fountain, the *Revealed Word*, consulting second hand authorities; all fallible, none unprejudiced.

*The Bible, the Text Book,—the Parent, the Teacher*, this must be the agency which men will at last come to; this is the most practicable scheme for human amelioration. A dollar will buy the Bible; the teachings cost nothing, the compensation being that sweet love, that pure enjoyment, that delicious intercommunion, which goes out and back from parent to child in such an occupation. With such instructions, in brief instalments, given kindly, lovingly, patiently, the daughter never takes to the street, nor the son to the gutter; the sister grows up pure, the brother grows up manly; the "dishonorable transaction," the "clandestine marriage," the "unhappy match," never break up the peace, never wither the hopes, nor "blast the expectations" of such a domestic circle. It is not the children of this sort of folk who fill jails, people penitentiaries, crowd the hospitals and rend the air of asylums with the shrieks of insane occupants. No, no, no! the frequenters of the church, the lecture room, and the prayer meeting; children who have memorized the catechism, and attended the Bible class, who learned to sing the "psalms, and hymns, and spiritual songs" of the olden time; it is not from the company of these that annual drafts are made to replenish the ranks of the daily dying in the institutions just named. Recruits are furnished full fast from orphaned or neglected children, whose education is obtained in the street; who run to see all the "fights," pilfer from huckster's stands, crowd around the show door for the chance of "slipping in," who attend militia musters and target shootings, who save their chance dimes for the circus, the negro singer, and the theatre, who "pitch quoits" with copper cents, and play marbles for "keeps," who delight in the strains of "*Old Dan Tucker*, *Lucy Long*, and *Jim along Josey*;" who take to the cigar at ten years of age, and learn "to chew" even earlier; who can "take a glass" in the manner of a finished gentleman, and order an oyster supper for a friend from the country with the air of a millionaire, and all before the "teens" are passed; a little later, and lower down, comes the opera, (as now managed generally,) with plots founded on infidelity, debauchery and crime, with the club house, the Sunday afternoon "drive." By this time they learn to "discuss" questions, but never any higher up than the last play or novel, the new star or prima donna; in the absence of these they glorify free living, abnegate marriage, with low impertinences disparaging women, cursing the difficulties of divorce, and launching out in praises of free love. About this time the money is gone, if not sooner; "position" lost, and character questionable, while the sheriff's warrant for forgery, or a ticket to the hospital for infamous disease closes their public life; the short remnant of it still remaining, to be passed in the felon's cell, or as the occupant of "a bed," one among surrounding thousands, where the groans of the daily dying, and the shrieks of those in mortal agony, are merely interludes in the drama going on in the heart; the escapeless, the ceasless hugging of sharp-pointed memories.

Parents, the moral is to you. See to it, that your children's reading is that which is useful, substantial and true, and that all their recreations be healthful as to the body, and as to the heart, refining and elevating.

